# A novel approach with holmium laser ablation for endoscopic management of intrahepatic biliary stricture

**DOI:** 10.1186/s12876-019-1093-y

**Published:** 2019-11-01

**Authors:** Jianying Lou, Qida Hu, Tao Ma, Wei Chen, Ji Wang, Prasoon Pankaj

**Affiliations:** 1 Department of Hepatobiliary and Pancreatic Surgery, Second Affiliated Hospital, Zhejiang University School of Medicine, 88 Jiefang Rd, Hangzhou, 310009 Zhejiang China; 2 Department of Hepatobiliary and Pancreatic Surgery, First Affiliated Hospital, Zhejiang University School of Medicine, 79 Qingchun Rd, Hangzhou, 310003 Zhejiang China

**Keywords:** Holmium laser ablation, Cholangioscopy, Hepatolithiasis, Biliary stricture

## Abstract

**Background:**

Hepatolithiasis, featuring high incidence, severe symptoms, and common recurrence, poses a heavy disease burden. Endoscopic management provides an opportunity to cure hepatolithiasis, but fails to properly resolve biliary stricture without additional interventional techniques. An innovative approach towards endoscopic management of biliary stricture is required.

**Methods:**

Holmium laser ablation was applied to biliary strictures via endoscopic access. Patients’ demographic, operative, and follow-up data after receiving holmium laser ablation were retrospectively collected for analysis.

**Results:**

A total of 15 patients (4 males and 11 females) underwent stricture ablation by holmium laser via cholangioscopy. All the patients successfully received holmium laser ablation, indicating a technical success rate of 100%. No postoperative mortality or no major perioperative complication was observed. During the follow-up period, the recurrence-free rate was 73% at 2 years and 67% at 5 years.

**Conclusions:**

We successfully developed a novel technique of biliary stricture removal by cholangioscopic holmium laser ablation with satisfying clinical outcomes.

## Background

Hepatolithiasis is a common biliary disorder found in Asian population, with an incidence rate ranging from 2 to 25% in Southeastern Asia [1–4]. Hepatolithiasis, characterized by intrabiliary stone formation, results in bile stasis, and sometimes recurrent cholangitis. Patients suffering from the disease also bear high risk of stone recurrence even after surgical treatment, mainly due to unsolved etiological problems such as anatomical biliary stricture or transient portal bacteremia [5, 6]. Additionally, hepatolithiasis is also an established risk factor for cholangiocarcinoma with very poor prognosis [4]. Therefore, the high incidence, the severe symptoms, and the common recurrence altogether contribute to a heavy disease burden on the society and a low quality of life for the patients.

Current principles for hepatolithiasis management include stone clearance, biliary stricture removal, proper bile drainage, and resection of severely involved liver, aiming to reduce the postoperative recurrence rate [7]. A variety of methods have been practiced for clinical hepatolithiasis management. One major approach is hepatic resection, which simultaneously resolves the problems of intrahepatic stones and biliary stricture by en-bloc surgical removal [8, 9]. Hepatic resection could also benefit the patients with reduced risks of cholangiocarcinoma [10], because the technique allows for complete removal of the liver parenchyma with the potential of carcinogenesis. Another approach is endoscopic removal of intrahepatic stone, featured by minimally invasive lithotomy that significantly enhances postoperative recovery and reduces hospital stay [11, 12]. The endoscopic lithotomy procedure could be achieved by either choledochoscopy via an exploratory incision into the common bile duct or cholangioscopy via percutaneous transhepatic approach [6, 11–14]. In particular, the patients with recurrent hepatolithiasis, who might have complicated surgical history or insufficient liver remnant, are usually unable to tolerate another major hepatic resection due to severe adhesion or possible postoperative liver function failure. Endoscopic approach proposes an opportunity to cure hepatolithiasis for those “inoperable” patients, but cannot properly resolve biliary stricture without additional interventional techniques.

Currently, only a few methods are available for biliary stricture management, including biliary stent [15, 16], balloon dilation [17, 18], radiofrequency ablation [19], and biliary scraper [20]. However, the recurrent rate of hepatolithiasis remains high. Effective management strategy in endoscopic approach for biliary stricture is still required. Here we propose an innovative approach using a holmium:yttrium-aluminum-garnet (Ho:YAG) surgical laser, a widely used technique in lithotripsy, for stricture ablation and complete stone clearance in endoscopic lithotomy.

## Methods

### Operative procedures

An endoscope with a 7.5 F outer diameter and a 3.6 F functioning route (Olympus, Japan) was applied for percutaneous cholangioscopy via a T-tube tract or other available access to the biliary tree. Continuous warm 0.9% saline solution (34–38 °C) was flushed via the functioning route for proper distension of the biliary ducts and clear vision of the surgical field after debris elimination. In situation of an encountered biliary stricture, a 200 mm optical fiber was inserted into the functioning route to access the stricture site, with the other end connected to a 60 W solid-state holmium unit consisted of a VersaPulse PowerSuite holmium laser (Lumenis, Yokneam, Isreal) and a control module (ZTC, Xiamen, China). The laser beam frequency was set to 6–10 Hz, and the energy was set to 0.5–1.0 J.

**Fig. 1:**
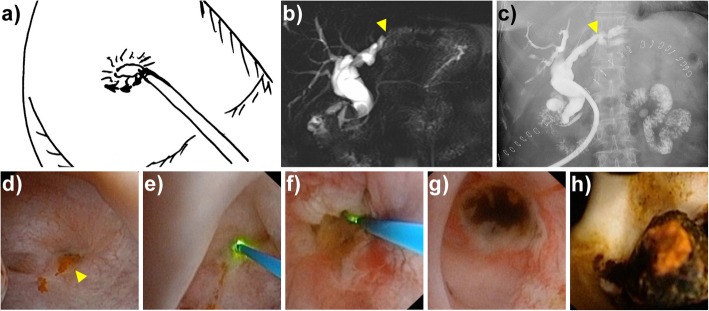
Holmium laser ablation for biliary stricture in a representative case, who had biliary stricture at the left hepatic duct. **a** A cartoon demonstration of biliary stricture ablation by laser ablation via T tube access. The arrows indicate the spiral cutting route in laser ablation. **b** Preoperative magnetic resonance cholangiopancreatography (MRCP) did not show the visualization of the distal biliary duct in the left lobe. **c** Postoperative T-tube cholangiography study showed visualization of the distal biliary duct. **d** The biliary stricture visualized during cholangioscopy. The yellow triangle indicates the stricture. **e** Initiation of holmium laser ablation at the stricture. **f** Holmium laser ablation in a spiral cutting manner. **g** Laser ablation accomplished, with relieved stricture allowing for cholangioscopy to pass through. **h** Multiple pigment stones observed in the distal biliary duct beyond the stricture location

We initiated stricture ablation using the Ho:YAG laser at the superficial inner surface of the biliary stricture, and carefully cut the stricture in a spiral manner within the depth of 1 mm (Fig. 1). The horizontal or transverse cutting, other than the spiral cutting, could eventually damage the adjacent vascular structure, which could lead to catastrophic consequences such as massive bleeding and should therefore be avoided. Mild biliary bleeding could be well controlled by holmium laser cautery using same beam energy and frequency as laser cutting. Active biliary bleeding should be managed with laser cautery at first, or be managed with prepared open hepatectomy to remove the bleeding lesions if unsuccessful with laser cautery. In most circumstances of stricture removal, the biliary stricture would loosen to let through cholangioscopy, after cutting the biliary stricture on less than 1/4 spiral of the inner surface. The reason might be that lysis of the fibrosis adhesion in part of the stricture tissue subsequently resulted in spontaneous lysis of the whole stricture. The cholangioscopy was then able to access the obstructed segment of the liver to continue the lithotripsy. The holmium laser ablation could be repeatedly applied to further biliary stricture until the biliary tree was free of stricture.

### Study design

We retrospectively reviewed the patients who underwent Ho:YAG laser stricture ablation from May 2012 to June 2015 at the Department of Hepatobiliary and Pancreatic Surgery, Second Affiliated Hospital, Zhejiang University School of Medicine. All the medical records from the electronic medical record system and the archived documents were used to collect demographic, operative, and follow-up data. Patients’ age, gender, stricture condition, procedure duration, and status of biliary stones were collected as variables of interests. Subsequent medical records after stricture ablation were analyzed to determine clinical success, follow-up duration, occurrence of stricture or stone recurrent. The patients were evaluated with ultrasound sonography after stone clearance. This study was approved by the institutional review board (approval number 2012–355). All the patients signed the informed consent before the procedure.

### Statistical analysis

Data are presented as mean ± standard deviation (s.d.). Analysis of variance or the nonparametric Kruskal-Wallis test was used to compare continuous factors, and the Pearson χ^2^ test was used for categorical variables. Recurrence-free survival analysis was performed to ascertain mean duration from stone clearance to recurrence, with Kaplan-Meier plots constructed. Statistical analyses were performed by Prism 7 (GraphPad, La Jolla, USA).

## Results

A total of 15 patients (4 males and 11 females) underwent stricture ablation by Ho:YAG laser via cholangioscopy (Table 1). Among them, 12 patients were referred from the local medical centers, and the other 3 patients were directly admitted to our center. The average age of the patients was 58 ± 9 years old (mean ± s.d.; median 58, range 41–70). All patients had biliary stricture with multiple intrahepatic stones. These patients were initially diagnosed as biliary stones at 47 ± 14 years old (mean ± s.d.; median 52, range 23–64), and progressed to biliary stricture requiring operation in 11 ± 8 years (mean ± s.d.; median 10, range 1–30).

**Table 1:** Demographic characteristics of included patients

Case #	Gender	Age (years old)	Age of initial hepatolithiasis diagnosis (years old)	Previous surgical history
1	Male	63	43	None
2	Female	70	60	(1) Open cholecystectomy (2) open common bile duct exploration (CBDE)
3	Female	53	23	(1) Open cholecystectomy & CBDE (2) open left lateral hepatectomy & CBDE
4	Male	65	60	(1) Open cholecystectomy & CBDE (2) open left hemihepatectomy & CBDE
5	Male	58	52	(1) Open cholecystectomy & CBDE (2) ERCP with stone removal
6	Female	66	64	Open cholecystectomy & CBDE
7	Female	68	58	(1) Open cholecystectomy (2) open CBDE
8	Female	56	41	(1) Open cholecystectomy (2) open CBDE
9	Female	65	52	(1) Open cholecystectomy (2) open CBDE
10	Female	67	61	Open cholecystectomy
11	Female	41	31	Open cholecystectomy & CBDE
12	Female	58	38	(1) Open cholecystectomy (2) open CBDE
13	Male	48	38	Open cholecystectomy, CBDE, left lateral hepatectomy & hepaticojejunostomy
14	Female	41	26	(1) Open cholecystectomy (2) open CBDE
15	Female	58	57	None

*ERCP* endoscopic retrograde cholangiopancreatography

The diagnosis of intrahepatic biliary stricture in all patients was confirmed during the operation. The 15 cases were all presented with multiple pigment stones, among which 5 cases (33%) had choledocal stones (Table 2). All the cases presented single biliary stricture, with 7 cases in the left lobe, 7 cases in the right lobe mainly in segments VI and VII, and 1 case in the confluent biliary duct. All the patients successfully received Ho:YAG laser ablation of biliary stricture via choledocoscopy or cholangioscopy via routes of previously placed T tube (10 cases), open operation (4 cases), or laparoscopic access (1 case), with a technical success rate of 100%. The total operation time for the initial cholangioscopy with stricture ablation was 47 ± 12 min (mean ± s.d.; range 25–65). Specifically, the Ho:YAG ablation procedure took the surgeons only 25 ± 11 s (mean ± s.d.; range 12–44) to open the stricture. The residual stones were then eliminated by subsequent cholangioscopic lithotripsy or stone removal procedures with a median time of 2 sessions (range was no postoperative mortality or major perioperative complication such as biliary hemorrhage or liver failure. Minor discomforts in patients were observed: one patient developed abdominal distension with temporary fever due to incomplete ileus; one patient had fever with the maximum body temperature of 38.3 °C; another patient had diarrhea 3 days post the procedure. These three patients were well managed with conservative treatment and finally discharged.

**Table 2:** Characteristics of biliary stricture and perioperative parameters

Case #	Location of the biliary stricture	Location of hepatolithiasis	Access route of the cholangioscopic stricture ablation	Duration of the initial cholangioscopy (mins)	Operative duration of Ho:YAG laser ablation (secs)	Subsequent cholangioscopy sessions	Major postoperative complications
1	Sgts II-III	Bilateral lobes & CBD	Laparoscopic	45	12	4	No
2	Sgts II-III	Bilateral lobes	T tube	60	20	4	No
3	Sgts VI-VII	Bilateral lobes & CBD	T tube	50	32	0	No
4	Right hepatic duct	Right lobe & CBD	T tube	55	12	3	No
5	Sgts II-III	Bilateral lobes	T tube	50	12	3	No
6	Left hepatic duct	Left lobe	T tube	50	34	2	No
7	Left hepatic duct	Left lobe	T tube	60	40	2	No
8	Sgts VI-VII	Bilateral lobes & CBD	T tube	55	19	8	No
9	Bile duct confluence	Bilateral lobes	Open	25	27	3	No
10	Right hepatic duct	Sgts VI-VII	Open	30	14	1	No
11	Sgts II-III	Bilateral lobes	T tube	65	36	6	No
12	Sgt VI	Sgt VI	Open	35	44	1	No
13	Sgt VII	Right lobe	T-tube	35	33	2	No
14	Sgts II-III	Sgts II-III	Open	40	19	1	No
15	Sgts VI-VII	Right lobe	T tube	50	25	2	No

*CBD* common bile duct, *Sgt* segment

**Fig. 2:**
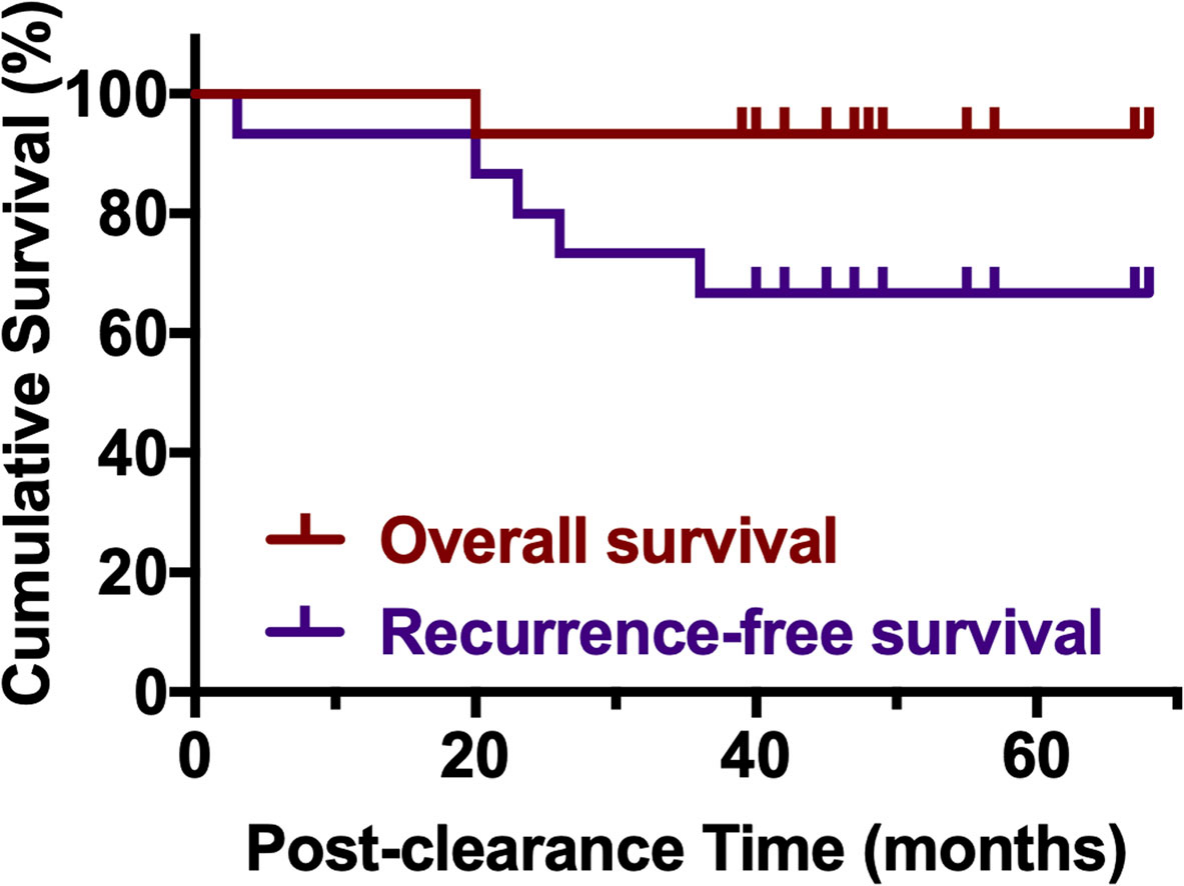
Overall (red) and recurrence-free (purple) survival of patients post laser stricture ablation and stone clearance via cholangioscopies

All patients were then followed up for 48 ± 12 months (mean ± s.d.; median 47, range 20–68) to obtain the long-term outcome data (Table 3). Four patients had hepatolithiasis recurrence during follow-up. A 63-year-old male had a rapid stricture recurrence with multiple stones at the left lateral lobe after 3 months post initial intervention, and underwent open left lateral hepatectomy, but unfortunately developed pancreatic cancer at 36 months post stone clearance. Another 3 patients had hepatolithiasis recurrence at 23, 26, and 36 months post stone clearance, and all chose to receive conservative therapy other than surgical treatment. The other patients did not report any postoperative recurrence of hepatolithiasis or biliary stricture after stone clearance during the follow-up period, resulting in a recurrence-free rate of 73% at 2 years and 67% at 5 years (Fig. 2). Two patients did not have hepatolithiasis recurrence but suffered from severe cirrhosis and pancreatitis, respectively. The patient with severe cirrhosis had a background of secondary biliary cirrhosis due to long-term of bile duct stricture at the procedure, and passed away due to variceal bleeding at 20 months post stone clearance. No other patient mortality was observed, indicating a 5-year overall survival rate of 93% (Fig. 2).

**Table 3:** Long-term outcomes post laser ablation of biliary stricture

Case #	Follow-up duration (months)	Recurrence of hepatolithiasis (time post stone clearance)	Recurrence of biliary stricture (time post stone clearance)	Other long-term outcomes (time post stone clearance)	Overall survival (months)	Recurrence-free survival (months)
1	47	Yes, multiple stones at left lateral lobe (3 months)	Yes (3 months)	Pancreatic cancer (36 months)	47	3
2	42	No	No	No	42	42
3	67	No	No	No	67	67
4	48	Yes, multiple stones at right lobe and CBD (26 months)	No	No	48	26
5	49	No	No	No	49	49
6	57	No	No	No	57	57
7	46	No	No	No	45	45
8	68	No	No	No	68	68
9	40	No	No	No	40	40
10	47	No	No	No	47	47
11	20	No	No	Cirrhosis-associated death (20 months)	20	20
12	55	No	No	Pancreatitis (30 months)	55	55
13	39	Yes, multiple CBD stones (23 months)	No	No	39	23
14	46	No	No	No	45	45
15	48	Yes, multiple CBD stones (36 months)	No	No	48	36

*CBD* common bile duct

## Discussions

In this study we presented a novel method of hepatoliathiasis treatment to eliminate biliary stricture by Ho:YAG laser ablation during cholangioscopy. During the follow-up period of 717 person years, our study cohort demonstrated a superior recurrence-free rate, and 2/3 of the cohort individuals did not encounter stone recurrence. These results suggest that holmium laser ablation could be an effective approach for biliary stricture removal.

The holmium laser ablation is indicated in several clinical settings. The most common indication is biliary stricture with distal duct stones discovered during cholangioscopic exploration. Inappropriate managements, such as leaving the stricture intact, would results in residual hepatolithiasis stones in distal biliary duct and rapid recurrence of newly formed stones, even if the proximal duct stones had been clear. An alternative approach is surgical resection of the liver with biliary stricture, but it may cause significant blood loss and severe postoperative complications, especially in the patients with complicated biliary surgery history. Another indication of the laser ablation is biliary duct recanalization from the distal end in percutaneous transhepatic cholangioscopy (PTCS), to restore natural biliary drainage intrahepatically and to reduce future recurrence. Untreated biliary strictures during transhepatic cholangioscopy would block the cholangioscope from further inspection of the common bile duct, and could eventually induce stone recurrence due to impaired biliary patency. Therefore, the holmium laser ablation, due to its advantage of minimal invasiveness, is now the first choice for recurrent biliary stricture in our hospitals, after the initial surgical treatments lead to massive adherence and fast recurrence.

Holmium laser delivers pulsatile high energy to fragment bilious stones, which could easily cut the stricture tissue [21, 22]. Based on our experience, some membrane-like biliary strictures could be easily managed with a single and short episode of holmium laser ablation, and the stricture will spontaneously relieve itself by biliary ductal flexibility. Other kinds of adherence biliary strictures require a longer working episode of holmium laser and a spiral maneuver to be completely eliminated.

Meanwhile, the prolonged working episode of holmium ablation might raise the concern of excessive injury and biliary hemorrhage. In a few cases, mild biliary hemorrhage could be well controlled by a further holmium laser hemostatsis at the same beaming frequency and energy as ablation. However, we have not encountered any case of uncontrolled active biliary bleeding yet. In our previous experience of other biliary surgery cases, temporarily blocking the entry route of cholangioscopy to increase the intraluminal pressure of intrahepatic biliary duct may help to stop uncontrolled biliary bleeding. Within the reach of cholangioscopy, biliary hemorrhage could also be managed by using biliary stent to create focal pressure on the bleeding site [23]. The crisis of uncontrolled biliary hemorrhage could at last be managed with interventional radiology or emergency open laparotomy as well.

Comparing to other techniques previously reported [15–20], the holmium laser ablation approach for biliary stricture shows several advantages. Firstly, the holmium laser ablation achieved a lower recurrence rate, which might benefit from more precise targeting, easier dilation, fewer scar formation, and less foreign substance retention in the biliary duct. Secondly, the holmium laser is originally designated for lithotripsy, and could be directly used for stricture ablation, therefore saving the surgeons’ time to change to new devices usually requiring additional learning. Finally, the shared device with lithotripsy also reduces the surgical cost on medical device, making the holmium laser ablation the most cost-effect approach among other techniques. Notably, the holmium laser ablation could be performed in combination with balloon dilation to accomplish better effect to relieve biliary stricture.

Our study also had several limitations. The first limitation is due to the study population, which had a small size and only included Chinese adult patients. The technique will be verified in a larger number of patients in the future, with homogeneous subgroups classified according to etiology, location, and number of the biliary stenosis. The second limitation is the follow-up duration, which is proper to evaluate the stone recurrence rate, but relatively short to allow better evaluation of late complications.

## Conclusions

In conclusion, we developed a novel technique of biliary stricture removal by cholangioscopic holmium laser ablation with satisfying clinical outcomes. The stricture removal allows for cholangioscopic lithotripsy in more endoscopic settings such as distal-to-proximal PTCS approach, broadening the minimally invasive applications of endoscopic interventions against hepatolithiasis. Since hepatolithiasis manifests as a chronic disease, future research should be focused on technique upgrades to persistently prevent stricture formation and to efficiently reduce hepatolithiasis recurrence during the long follow-up period.

## Data Availability

All data generated or analysed during this study are included in this article.

## Abbreviations

Ho:YAG: holmium:yttrium-aluminum-garnet
PTCS: percutaneous transhepatic cholangioscopy
s.d.: standard deviation
